# Attrition in longitudinal randomized controlled trials: home visits make a difference

**DOI:** 10.1186/1471-2288-12-178

**Published:** 2012-11-23

**Authors:** Janey C Peterson, Paul A Pirraglia, Martin T Wells, Mary E Charlson

**Affiliations:** 1 Division of Clinical Epidemiology and Evaluative Sciences Research, Weill Cornell Medical College, 1300 York Avenue, Box 46, New York, NY, 10065, USA; 2 Providence VA Medical Center and Alpert Medical School of Brown University, Providence, RI, USA; 3 Department of Statistical Science, Cornell University, Ithaca, NY, USA

**Keywords:** Loss to follow-up, Coronary artery bypass graft (CABG) surgery, Cardiovascular disease, Epidemiological methods, Dropouts, Non-response bias, Non respondents, Home visit, Predictors of attrition, Strategies to reduce attrition

## Abstract

**Background:**

Participant attrition in longitudinal studies can introduce systematic bias, favoring participants who return for follow-up, and increase the likelihood that those with complications will be underestimated. Our aim was to examine the effectiveness of home follow-up (Home F/U) to complete the final study evaluation on potentially “lost” participants by: 1) evaluating the impact of including and excluding potentially “lost” participants (e.g., those who required Home F/U to complete the final evaluation) on the rates of study complications; 2) examining the relationship between timing and number of complications on the requirement for subsequent Home F/U; and 3) determining predictors of those who required Home F/U.

**Methods:**

We used data from a randomized controlled trial (RCT) conducted from 1991–1994 among coronary artery bypass graft surgery patients that investigated the effect of High mean arterial pressure (MAP) (intervention) vs. Low MAP (control) during cardiopulmonary bypass on 5 complications: cardiac morbidity/mortality, neurologic morbidity/mortality, all-cause mortality, neurocognitive dysfunction and functional decline. We enhanced completion of the final 6-month evaluation using Home F/U.

**Results:**

Among 248 participants, 61 (25%) required Home F/U and the remaining 187 (75%) received Routine F/U. By employing Home F/U, we detected 11 additional complications at 6 months: 1 major neurologic complication, 6 cases of neurocognitive dysfunction and 4 cases of functional decline. Follow-up of 61 additional Home F/U participants enabled us to reach statistical significance on our main trial outcome. Specifically, the High MAP group had a significantly lower rate of the Combined Trial Outcome compared to the Low MAP group, 16.1% vs. 27.4% (p=0.032). In multivariate analysis, participants who were ≥ 75 years (OR=3.23, 95% CI 1.52-6.88, p=0.002) or on baseline diuretic therapy (OR=2.44, 95% CI 1.14-5.21, p=0.02) were more likely to require Home F/U. In addition, those in the Home F/U group were more likely to have sustained 2 or more complications (p=0.05).

**Conclusions:**

Home visits are an effective approach to reduce attrition and improve accuracy of study outcome reporting. Trial results may be influenced by this method of reducing attrition. Older participants, those with greater medical burden and those who sustain multiple complications are at higher risk for attrition.

## Background

Attrition in longitudinal randomized controlled trials (RCTs) is a challenge faced by every clinical investigator. In studies of medical and surgical patients, participants who are lost to follow-up have distinct characteristics, including worse functional outcomes, new complications or death [1-3]. It has been suggested that attrition of 5% or less is unlikely to introduce bias, and conversely, attrition rates of 20% or more raise concerns about validity [4]. In a review of RCTs reporting time to event outcomes in 4 leading medical journals, only 44% published detailed and consistent loss to follow-up information [5]. A review that examined missing outcome data in RCTs published in 4 major medical journals found that 89% of studies had some missing outcome data and 18% of studies had more than 20% of participants with missing outcome data [6]. Some journals will not publish RCTs with attrition that exceeds 20% [7]. Attrition is therefore a major concern for RCTs as it raises important methodological questions, such as, what was the clinical course of participants who are missing or have dropped out and what would be the effect if missing or “lost” participants could be located and included in the reporting of study outcomes (i.e., complication rates)?

Study attrition creates bias in the direction of those who complete participation in longitudinal studies. Complications such as mortality are found only with intensive efforts focused on obtaining complete follow-up, and the complication rate for a group of participants can dramatically shift when potentially lost participants are ultimately located and included in the analysis [2]. Thus, when high attrition rates are present, there is the likelihood that those participants with complications have been underestimated. In a longitudinal study of patients who had undergone total hip replacement, Murray and colleagues found that participants lost to follow-up had significantly worse pain, less movement, as well as worse progress and radiological scores at their last recorded visit when compared to participants who completed follow-up [8].

Since the late 1980’s, there has been great interest in neurocognitive complications following cardiac surgery. Over the intervening years, percutaneous coronary interventions (e.g., angioplasty, stenting and minimally invasive procedures) have grown in popularity; traditional cardiac surgical revascularization is now performed less frequently, and reserved for those with diffuse triple vessel or left main disease, particularly in the setting of diabetes [9]. One reason why Home F/U is particularly important is that other cardiac surgery studies that have assessed longitudinal neurocognitive functioning over 6–12 months post-operatively have reported loss to follow-up ranging from 25-44% [10-12]. To our knowledge, no other studies have focused on increasing follow-up in cardiac surgery studies in general, and on improving neurocognitive follow-up, in particular.

Our study had 3 objectives: 1) to evaluate the impact of including and excluding potentially “lost” participants (e.g., those who required Home F/U in order to complete the final 6-month study evaluation) on the rates of reported study complications; 2) to examine the relationship between the timing and number of complications and the requirement for Home F/U; and 3) to determine predictors of participants who required Home F/U.

## Methods

Participants were primary elective CABG surgery patients who had participated in a prospective RCT examining the impact of intraoperative hemodynamic management on major cardiac and neurologic morbidity/mortality, all-cause mortality, neurocognitive dysfunction and deterioration in functional status [13]. Eligible participants were those scheduled to undergo elective or urgent multi-vessel CABG for left main or multi-vessel coronary artery disease. We have previously described the details of eligibility [13]. All participants provided informed written consent. We enrolled 248 participants between 1991 and 1994. We previously reported the RCT results with an intention-to-treat analysis [13]. The protocol, “Improving Outcomes and Quality of Life after CABG” (IRB # 0689–579) was approved by the Weill Cornell Institutional Review Board (IRB) in 1991 and the protocol has remained active and approved. The data are held in the Division of Clinical Epidemiology and Evaluative Sciences Research at Weill Cornell Medical College.

### Baseline, peri- and post-operative evaluations

The baseline evaluation has been reported elsewhere [13]. In brief, prior to surgery, we collected demographic and clinical characteristics of the participants by interview, including their clinical history, medications and the Charlson Comorbidity Index [14]. All participants received standardized baseline cardiologic and neurological examinations. We assessed neurocognitive function with an eleven-test neuropsychologic battery (WAIS-R Digit Span, Trail Making A and B, Boston Naming, Benton Visual Retention and Recognition Test, Controlled Oral Word Association, WAIS-R Digit Symbol, Mattis-Kovner Verbal Recall and Recognition and Finger Tapping Test). The Ammons Quick Test [15] was used as a proxy for verbal IQ. We also collected laboratory and electrocardiographic data.

We followed participants peri- and post-operatively according to a standardized surveillance protocol. Research assistants were present in the operating room and recorded all events (e.g., blood pressures, duration of cardiopulmonary bypass and number of bypass grafts). We have previously reported details of the anesthetic protocol [16]. The study cardiologist and neurologist, who were blinded to the intra-operative treatment protocol, performed standardized evaluations at 1, 2 and 7 days post-operatively (e.g., duration of endotrachial intubation, amount of blood loss and length of stay) and at 6 months. Final study complications (i.e., main trial outcomes) were assessed at 6 months.

We now present a secondary data analysis of this RCT according to method of follow-up (Routine F/U vs. Home F/U) required to complete the 6-month final evaluation. The 5 trial complications (i.e., main trial outcomes) were: 1) cardiac morbidity and mortality (i.e., myocardial infarction, low flow state/cardiogenic shock, cardiopulmonary arrest, adult respiratory distress syndrome and pulmonary edema); 2) neurologic morbidity and mortality (i.e., major focal deficit); 3) all-cause mortality; 4) neurocognitive dysfunction (i.e., deterioration in ≥ 3 tests in the 11 test neurocognitive battery); and 5) functional decline (i.e., > 5 point decline in the physical component summary measure of the SF-36 Health Survey [17-19]). We have previously described these complication definitions in detail [13].

With regard to power in the original RCT, we had estimated that the combined incidence of cardiac morbidity/mortality, neurologic morbidity/mortality, all-cause mortality, cognitive dysfunction and deterioration in functional status would be 35%. To compensate for the multiple complications without inflating the Type I error, a conservative Bonferroni correction (0.005) was applied. A drop in the incidence of any of the complications (delta) of 0.20 constituted a clinically important difference between the High and Low MAP strategies. Based on these estimated values of incidence, along with the specified delta and alpha with 80% power, the sample size estimate was 248 participants, or 124 in each group. We sought to enroll a sufficient number of participants to achieve 6-month follow-up on this sample size.

### Randomization arms

Participants were randomized to one of two strategies of hemodynamic management during cardiopulmonary bypass. In the control group, we maintained participants’ mean arterial pressure (MAP) during cardiopulmonary bypass at 50–60 mm Hg (usual care, “Low MAP”). In the experimental group, we increased MAP during cardiopulmonary bypass to 80–100 mm Hg (“High MAP”).

### Final study evaluation at 6 months

At 5 months, we contacted participants and asked them to return to the hospital for the final study evaluation (Routine F/U approach).

#### Routine F/U

We accommodated participant preferences, including evening and weekend appointments, and reimbursed all travel-related expenses. At this evaluation, we assessed participants for interval clinical events using a standardized set of questions, the blinded assessors performed standardized cardiac and neurological exams, and neurocognitive tests and questionnaires were re-administered (e.g., SF-36 Health Survey). We also obtained an electrocardiogram.

#### Home F/U

For participants who refused to schedule a Routine F/U appointment or who missed at least two scheduled appointments, we offered Home F/U. Home F/U consisted of the same study personnel traveling to the participant’s home or office to perform the study evaluation. To minimize time outside of the office, we grouped the visits by geographic location. After the visit, we contacted the patient’s physician to obtain a copy of the participant’s most recent electrocardiogram.

### Statistics

We used SAS (SAS 9.2, Cary, NC) for data analysis. Counts and percentages were calculated for the Routine F/U and Home F/U participants. For continuous variables (e.g., age, ejection fraction and the neurocognitive tests), we calculated means and standard deviations. For highly skewed data, we calculated the median and range. Variables such as age and the Charlson Comorbidity Index were categorized according to clinically important thresholds [20]. To evaluate difference between groups, we compared continuous variables with Student’s *t*-test, proportions using chi-square or Fisher’s exact test and ordinal or highly skewed data with the Wilcoxon rank-sum test. We also employed multivariate logistic regression and assessed the adequacy of the models using a Hosmer and Lemeshow type residual analysis [21].

Objective 1: In order to evaluate the impact of including and excluding potentially “lost” participants on the reporting of study complication rates, we first analyzed study complication rates by randomization group (High vs. Low MAP) in Routine F/U participants only. Had only Routine F/U been performed, study surveillance at days 1, 2 and 7 would have been conducted on all 248 participants and surveillance at 6 months would have been completed on the 187 participants who returned for Routine F/U. Five participants sustained early complications and were not in the Routine F/U group, bringing the total n to 192 for Routine F/U. To evaluate differences between the groups, we compared proportions using chi-square or Fisher’s exact test.

For all analyses of complications, each patient was counted only once. That is, for any of the 5 complications (cardiac complications, neurologic complications, all-cause mortality, neurocognitive dysfunction and functional decline) each patient was counted as meeting each complication criteria in a dichotomous +/− fashion. For example, if a patient sustained more than 1 cardiac complication, we counted the patient as meeting the criteria only once. In addition, in the final analysis, if a patient experienced any of the 5 complications, they were counted as experiencing the main study outcome (i.e., “Combined Trial Outcome”) in a dichotomous +/− fashion. Thus, in the main trial analysis, no patient was counted twice.

We next analyzed the same trial results (High vs. Low MAP) with the combined cohort: both the Routine F/U and Home F/U participants. This evaluation included all 248 participants in the early evaluation and 237 participants who completed the late, 6-month evaluation. Again, to evaluate differences between the randomization groups, we compared proportions using chi-square or Fisher’s exact test.

Objective 2: To examine the timing of complications relative to the need for Home F/U, we compared the rates of early and late complications in the Routine F/U and Home F/U participants. Specifically, we evaluated: 1) combined early cardiac and neurologic morbidity and mortality; 2) combined late (6-month) cardiac and neurologic morbidity and mortality; and 3) the overall rate of combined cardiac and neurologic morbidity and mortality between the 2 F/U groups. We used chi-square or Fisher’s exact test to examine proportions. Finally, we examined the rate of major cardiac/neurologic complications in the High MAP group in the Routine F/U and Home F/U participants and the rate of major cardiac/neurologic complications in the Low MAP group in the Routine F/U and Home F/U participants with chi-square and Fisher’s exact test. To examine whether number of complications was related to the need for Home F/U, we first tested the distribution of the number of complications (range 0–3) over 6 months in the Routine F/U and Home F/U groups using the Wilcoxon rank-sum test. We next examined the distribution of the number of complications in each F/U group by randomization (High vs. Low MAP). Finally, we evaluated the proportion of patients who sustained 2 or more complications by F/U group using the mid-p exact test [22].

Objective 3: To determine predictors of participants who required Home F/U, we employed multivariate logistic regression. We evaluated various models to assess the existence of possible interactions using logistic regression and model fit statistics. We employed multivariate logistic regression with Home F/U as the dependent variable, controlling for baseline comorbidity, (natural log) transformed hospital length of stay and randomization group. In all analyses, we counted the number of participants with events, not the number of events. No data were imputed.

## Results

The study population consisted of 248 participants. Overall, 61 participants (25%) required Home F/U to complete the 6-month evaluation. The remaining 187 participants (75%) received Routine F/U; 169 participants (68%) returned to the hospital to complete the 6-month evaluation, 7 (3%) died and 11 (4%) were lost.

### Baseline and peri-operative characteristics

Table 1 shows the baseline demographic and clinical characteristics for Routine F/U and Home F/U patients according to randomization group (Low vs. High MAP). Overall, the population had a mean age of 66 years and was predominantly Caucasian. Over 70% were married and over 47% had completed college. Participants reported having cardiovascular disease for an average of 5.2 years, 43% had a history of a previous myocardial infarction and 20% had a history of diabetes mellitus. There were baseline differences between the Routine F/U and Home F/U participants. Specifically, Home F/U participants were more likely to be 75 years or older (p=0.006) and receiving diuretic therapy (p=0.04), however, 70% of those on diuretics did not have congestive heart failure (CHF). In addition, Home F/U participants had significantly lower scores at baseline on the Ammons IQ test (p=0.04), Benton recognition (p=0.02), Trails B (p=0.04) and Digit Symbol (p=0.04) tests.

**Table 1:** Baseline demographic and clinical characteristics for Routine F/U and Home F/U patients according to randomization group in the original RCT

	**ROUTINE F/U, N=187**			**HOME F/U, N=61**		
	**% unless otherwise indicated**			**% unless otherwise indicated**		
	**ALL N=187**	**LOW MAP N=97 (52%)**	**HIGH MAP N=90 (48%)**	**ALL N=61**	**LOW MAP N=27 (44%)**	**HIGH MAP N=34 (56%)**
**Demographic**						
Age, mean years (SD)	65.2 (9.3)	65.2 (10.1)	65.3 (8.4)	67.7 (9.5)	70.0 (9.5)	65.9 (9.3)
75 years and older	13.9	16.5	11.1	29.5	40.7	20.6
Male	78.6	78.3	78.9	83.6	78.8	88.2
Caucasian	95.6	94.6	96.6	98.3	96.3	100
College education or higher	48.4	50.5	46.0	44.8	41.7	47.1
Married	73.3	73.2	73.3	77.0	63.0	88.2*
**Cardiac and Neurologic History**						
Cardiac symptom duration, mean years (SD)	5.0 (7.1)	5.3 (7.6)	4.7 (6.7)	5.7 (8.6)	5.6 (8.4)	5.9 (8.8)
Current and former smokers	72.7	71.1	74.4	78.7	81.5	76.5
Canadian cardiovascular class						
I	18.7	19.6	17.8	13.1	14.8	11.8
II	22.5	24.7	20.0	27.9	25.9	29.4
III	12.3	11.3	13.3	16.4	29.6	5.9
IV	27.8	30.9	24.4	16.4	14.8	17.7
Ejection Fraction, mean (SD)	48.5 (12.1)	49.3 (12.1)	47.6 (12.2)	47.4 (13.9)	47.2 (15.1)	47.6 (13.0)
Left main disease	13.9	14.4	13.3	11.5	22.2	2.9*
Previous MI	42.3	36.1	48.9	44.3	44.4	44.1
Congestive heart failure	10.7	10.3	11.1	11.5	14.8	8.8
COPD/Asthma	10.7	6.2	15.6*	6.6	7.4	5.8
Peripheral vascular disease	21.9	23.7	20.0	16.4	22.2	11.8
CVA	11.8	8.3	15.6	8.2	7.4	8.8
Charlson Comorbidity Score						
0-1	58.8	58.8	58.9	54.1	48.1	58.8
2-3	27.8	27.8	27.8	36.1	44.4	29.4
≥ 4	13.4	13.4	13.3	9.8	7.4	11.8
Hypertension	49.2	43.3	55.6	59.0	59.3	58.8
Dialysis	1.6	2.1	1.1	0	0	0
Renal dysfunction	5.9	6.2	5.6	4.9	3.7	5.9
Diabetes	20.3	23.7	16.7	19.7	18.5	20.6
End organ damage	44.7	43.5	46.7	33.3	40.0	28.6
**Medications**						
Diuretics	16.0	16.5	15.6	27.9	33.3	23.5
Calcium channel blockers	62.6	60.8	64.4	63.9	55.6	70.6
Nitrates	55.1	60.8	48.9	52.5	70.4	38.2*
Beta blockers	57.8	55.7	60.0	52.5	48.2	55.9
Ace inhibitors	16.6	17.5	15.6	16.4	22.2	11.8
**Neurocognitive tests**						
Ammons IQ Test, mean (SD)	106.6 (14.4)	107.2 (15.5)	106.0 (13.3)	102.3 (12.2)	100.2 (10.5)	103.9 (13.2)
*Linguistic Function*						
Boston Naming Test, median (range)	26.0 (8–30)	26.0 (8–30)	26.0 (11–30)	25 (9–30)	25 (12–30)	25 (9–30)
Controlled Word Association, mean (SD)	38.4 (13.4)	38.4 (13.5)	38.4 (13.3)	35.1(12.6)	34.2 (11.4)	35.7 (13.5)
*Memory*						
Benton Visual Recall, mean (SD)	5.2 (2.2)	5.3 (2.1)	5.1 (2.3)	4.6 (2.2)	5.0 (1.9)	4.3 (2.4)
Benton Recognition, median (range)	8 (3–10)	8 (3–10)	8 (4–10)	7.5 (0–10)	7 (3–10)	8 (0–10)
Mattis-Kovner Recall, mean (SD)	10.7 (3.4)	10.9 (3.4)	10.6 (3.4)	9.9 (3.6)	9.6 (4.0)	10.3 (3.2)
Mattis-Kovner, mean (SD)	2.7 (0–3.9)	2.7 (0–3.9)	2.7 (0–3.9)	2.6 (1.2-3.9)	2.4 (1.3-3.9)	2.7 (1.2-3.9)
Recognition, median (range)						
*Psychomotor function*						
Trails A, median (range)	40 (15–162)	38.5 (15–162)	41 (20–141)	39 (17–217)	39 (19–100)	41.5 (17–217)
Trails B, median (range)	86 (32–350)	85.0 (32–350)	89.5 (42–320)	104.5 (38–405)	117 (42–405)	92 (38–309)
Digit Symbol, mean (SD)	42.1 (12.3)	42.7 (13.6)	41.5 (10.7)	38.2 (11.9)	34.7 (12.3)	41.2 (10.9)*
Digit Span, mean (SD)	14.7 (4.1)	15.2 (4.0)	14.1 (4.1)	14.0 (3.7)	13.3 **(**3.5)	14.5 (3.8)
Finger Tapping Dominant, mean (SD)	47.1 (10.6)	47.0 (10.6)	47.2 (10.6)	44.2 (8.4)	45.0 (8.9)	43.5 (8.1)
Finger Tapping Non-dominant, mean (SD)	42.5 (8.9)	42.1 (9.2)	43.1 (8.7)	40.7 (8.1)	42.0 (8.0)	39.6 (8.2)

*Indicates comparison of High vs. Low MAP p<0.05.

With respect to differences in baseline characteristics between the Routine F/U and Home F/U patients according to randomization group (Low vs. High MAP) (Table 1), participants who received Routine F/U and were randomized to High MAP were more likely to have COPD or asthma (p<0.04) when compared to those randomized to Low MAP. Participants who received Home F/U and were randomized to High MAP group were more likely to be married (p=0.03), take nitrates (p=0.01), be free of left main disease (p=0.04) and score higher on the Digit Symbol test (p=0.04) when compared to those randomized to Low MAP.

We also evaluated peri- and post-operative characteristics to see if there were differences between the Routine F/U and Home F/U participants. The groups were similar with respect to number of bypass grafts placed during surgery, duration of time on cardiopulmonary bypass, duration of endotracheal intubation, amount of early post-operative blood loss and overall hospital length of stay (Table 2). When we examined the peri-operative characteristics in the Routine F/U and Home F/U participants according to randomization group (Low vs. High MAP) participants in the Routine F/U group who were randomized to High MAP received fewer bypass grafts during surgery (p=0.02) and had less blood loss in the initial 24 hours after surgery (p=0.03) (Table 2). There were no such differences noted between the Low and High MAP groups in the Home F/U participants.

**Table 2:** Perioperative characteristics for Routine F/U and Home F/U patients according to randomization group in the original RCT

	**ROUTINE F/U, N=187**			**HOME F/U, N=61**		
	**All**	**Low MAP N=97**	**High MAP N=90**	**All**	**Low MAP N=27**	**High MAP N=34**
Number of grafts, mean (SD)	3.0 (0.8)	3.1 (0.9)	2.9 (0.8)*	3.0 (0.8)	3.0 (0.8)	3.1 (0.8)
Time on cardiopulmonary bypass, mean minutes (SD)	89.1 (30.1)	92.7 (31.4)	85.1 (28.2)	81.2 (29.0)	77.2 (29.4)	84.3 (28.7)
Duration of endotrachial intubation, mean hours (SD)	29.2 (96.3)	22.0 (10.7)	37.2 (139.5)	25.4 (27.0)	32.1 (38.9)	19.8 (7.1)
Post-operative blood loss initial 24 h post-operatively, mean milliliters (SD)	980 (667)	1070 (769)	855 (521)*	892 (389)	803 (293)	962 (442)
Hospital length of stay, (median days, range)	11.0 (4-93)	11.0 (5–93)	11.0 (4–89)	10.0 (6-35)	11.0 (6–35)	10.0 (8–21)

*Indicates comparison of High vs. Low MAP p<0.05.

### Main trial complication rates

Displayed in Table 3 are the rates of peri-operative and 6-month cardiac and neurologic complications, neurocognitive dysfunction, functional decline and the Combined Trial Outcome when only the Routine F/U participants were considered. Table 4 displays the RCT trial results with the inclusion of both the Home F/U and Routine F/U groups. There are several notable differences between Table 3 and Table 4. First, as a result of conducting Home F/U, we were able to document 1 new case of major neurologic morbidity, 6 additional cases of neurocognitive dysfunction and 4 additional cases of functional decline (n=11) (Table 4). Overall, this resulted in 4 additional Combined Trial Outcomes, given that a single participant may have sustained multiple complications (Table 4). Second, and more importantly, including Home F/U participants enabled us to reach statistical significance in our Combined Trial Outcome, with the High MAP group having a significantly lower rate of complications when compared to the Low MAP group, 16.1% vs. 27.4% (p=0.032) (Table 4). In contrast, when we conducted this same analysis in Routine F/U participants only, the analysis did not reach statistical significance: 19.8% vs. 31.7% (p=0.061) (Table 3).

**Table 3:** Trial outcomes in Routine F/U patients

**CARDIAC AND NEUROLOGIC MORBIDITY AND MORTALITY (N=192)**		**P**	**# OF COMPLICATIONS**	
**Low MAP (n=101)**	15.8%		Perioperative	13
			6 months	4
			**Total**	17
		0.022		
**High MAP (n=91)**	5.5%		Perioperative	4
			6 months	3
			**Total**	5
**Total Cardiac and neurologic morbidity and mortality**	10.9%		**Total**	21
**Neurocognitive Dysfunction Cardiac and Neurologic Morbidity and Mortality at 6 months (n=166)**				
Low MAP	11.6%	0.68	6 months	10
High MAP	13.8%		6 months	11
**Total**	12.7%			21
**Functional Decline at 6 months n=160)**				
Low MAP	9.4%	0.38	6 months	8
High MAP	5.3%		6 months	4
**Total**	7.5%			12
**Combined Trial Outcome (n=192)**				
Low MAP	31.7%	0.061		32
High MAP	19.8%			18
**Total**	26.0%			50

(Surveillance on 248 participants perioperatively and 187 participants at 6 months).

**Table 4:** Trial outcomes in Routine F/U and Home F/U patients

**CARDIAC AND NEUROLOGIC MORBIDITY AND MORTALITY (N=248)**		**P**	**# OF COMPLICATIONS**	
**Low MAP (n=124)**	12.9%		Perioperative	13
			6 months	4
			**Total**	17
		0.026		
**High MAP (n=124)**	4.8%		Perioperative	4
			6 months	4
			**Total**	6
**Total Cardiac and neurologic morbidity and mortality**	8.9%		**Total**	22
**Neurocognitive Dysfunction at 6 months (n=225)**				
Low MAP	12.4%	0.86	6 months	14
High MAP	11.6%		6 months	13
**Total**	12.0%			27
**Functional Decline at 6 months (n=217)**				
Low MAP	8.3%	0.62	6 months	9
High MAP	6.5%		6 months	7
**Total**	7.4%			16
**Combined Trial Outcome (n=248)**				
Low MAP	27.4%	0.032		34
High MAP	16.1%			20
**Total**	21.8%			54

(Surveillance on 248 participants perioperatively and 237 participants at 6 months).

MAP= Mean Arterial Pressure. Combined Trial Outcomes included the following complications at 6 months: cardiac morbidity (myocardial infarction, pulmonary edema, adult respiratory distress syndrome and cardiogenic shock); neurologic morbidity (major focal deficit); all-cause mortality; functional decline (>5 point decline in physical component summary measure of the SF-36); and neurocognitive deterioration (within-patient differences on an 11 test neurocognitive battery).

### Timing of complications

We next assessed the timing of complications to evaluate whether complications were associated with the need for a Home F/U. As shown in Table 5, most major cardiac and neurologic complications occurred peri-operatively and the rates of early and late complications did not differ between the 2 F/U groups. Specifically, the rate of peri-operative cardiac and neurologic complications was 6.4% vs. 8.2% (p=0.82) and late complications (6 months) was 2.7% vs. 4.9% (p=0.62) in Routine F/U vs. Home F/U participants, respectively. The overall rate of combined (early + late) cardiac and neurologic complications was also similar in the 2 F/U groups: 8.6% (16/187) in the Routine F/U group compared to 9.8% (6/61) in the Home F/U group (p=0.76) (Table 5). We next evaluated whether there were differences in the rates of major cardiac and neurologic complications according to randomization group. The rates of major complications in the Low (Routine F/U 12.4% vs. Home F/U 14.8%) and High MAP groups (Routine F/U 4.4% vs. Home F/U 5.9%) did not differ according to F/U group (p=0.95 and p>0.99, respectively).

**Table 5:** Complications in Routine F/U and Home F/U patients according to randomization group

	**ROUTINE F/U, N=187**			**HOME F/U, N=61**		
**EVENT**	**All % (n)**	**Low MAP N=97, % (n)**	**High MAP N=90 % (n)**	**All % (n)**	**Low MAP N=27, % (n)**	**High MAP N=34, % (n)**
Perioperative cardiac and neurologic morbidity and mortality	6.4 (12)	9.3 (9)	3.3 (3)	8.2 (5)	14.8 (4)	2.9 (1)
6-month cardiac and neurologic morbidity and mortality	2.7 (5)	3.1 (3)	2.2 (2)	4.9 (3)	3.7 (1)	5.9 (2)
Total cardiac and neurologic morbidity and mortality	8.6 (16)	12.4 (12)	4.4 (4)	9.8 (6)	14.8 (4)	5.9 (2)
Functional decline (6 months)	7.5 (12)	9.4 (8)	5.3 (4)	7.0 (4)	4.2 (1)	9.1 (3)
Neurocognitive dysfunction (6 months)	12.7 (21)	11.6 (10)	13.8 (11)	10.2 (6)	14.8 (4)	6.3 (2)
Total Combined Outcome	24.0 (45)	28.9 (28)	18.9 (17)	14.8 (9)	22.2 (6)	8.8 (3)

Complications were cardiac morbidity (myocardial infarction, pulmonary edema, adult respiratory distress syndrome and cardiogenic shock); neurologic morbidity (major focal deficit); all-cause mortality; functional decline (>5 point decline in physical component summary measure of the SF-36); and neurocognitive deterioration (within-patient differences on an 11 test neurocognitive battery).

### Multiple Complications

We next evaluated the number of complications according to Routine F/U vs. Home F/U and randomization group (Table 6). The distribution of the number of complications at 6 months in the Routine F/U vs. Home F/U participants was similar (p=0.22). There were also no differences by randomization group. However, 2.1% of Routine F/U participants and 8.1% of Home F/U participants sustained 2 or more study complications (i.e., major cardiac or neurologic morbidity and mortality, all-cause mortality, neurocognitive dysfunction or functional decline), a significant difference (p=0.05) (Table 6). When we evaluated the combination of the most serious complications, an early major cardiac or neurologic complication *and* a new 6-month major cardiac or neurologic complication, 3 participants met this criteria; 2 in the High MAP group and 1 in the Low MAP group (data not displayed). Two of these participants were seen at 6 months with Home F/U and 1 with Routine F/U. Finally, 1 participant experienced combined early cardiac and neurologic complications. This participant required Home F/U at 6 months.

**Table 6:** Number of complications in Routine F/U and Home F/U patients according to randomization group in the original RCT

	**ROUTINE F/U N=187**		**HOME F/U N=61**	
**Number of complications**	**Low MAP, N=97 % (n)**	**High MAP N=90 % (n)**	**Low MAP N=27 % (n)**	**High MAP N=34 % (n)**
0	71.1 (69)	81.1 (73)	77.8 (21)	91.2 (31)
1	26.8 (26)	16.7 (15)	11.1 (3)	2.9 (1)
2	2.1 (2)	2.2 (2)	11.1 (3)	0
3	0	0	0	5.6 (2)

Complications=Major cardiac and neurologic morbidity/mortality + all cause mortality + neurocognitive dysfunction + functional decline.

### Predictors of Requiring Home F/U

In multivariate logistic regression, we evaluated predictors of requiring Home F/U at 6 months. Table 7 demonstrates the multivariate results, along with the odds ratio and 95% confidence intervals. Age 75 years or older (OR=3.23, 95% CI 1.52-6.88, p=0.002) and baseline diuretic therapy (OR=2.44, 95% CI 1.14-5.21, p=0.02) were both significant. The c statistic value (a point estimate of the area under a ROC curve) equal to 0.71 reveals that our model has good predictive ability.

**Table 7:** Multivariate model of predictors of Home F/U at 6 months*

	**Multivariate p**	**Odds ratio (95% Confidence Interval)**
**Baseline predictors**		
≥75 years of age	0.002	3.23 (1.52-6.88)
Pre-operative diuretic use	0.02	2.44 (1.14-5.21)
Ammons IQ	0.17	0.96 (0.96-1.01)
Early cardiac or neurologic morbidity	0.54	1.48 (0.42-5.20)

*Model adjusted for baseline comorbidity, (natural log) transformed hospital length of stay and randomization group.

## Discussion

The current study illustrates the importance of obtaining follow-up on participants who do not return for final evaluation in an RCT by displaying the effect of including Home F/U participants in the final analysis vs. not including them. By conducting Home F/U, we were able to detect an additional 11 complications: 1 new neurologic complication, 6 neurocognitive complications and 4 functional complications. With Home F/U, we achieved 6-month follow-up on an additional 61 participants (25%) and detected an additional 4 Combined Trial Outcomes. This resulted in statistical significance between the 2 randomization groups in the Combined Trial Outcome – Low MAP 27.4% vs. High MAP 16.1%, (p=0.032) (Table 4). To our knowledge, this is the first paper to report the effect of Home F/U on the main trial outcome (i.e., Combined Trial Outcome) in an RCT.

The implications of positive vs. negative trial results on clinical practice are significant. This RCT was a seminal study, and the first and only randomized study to test the efficacy of High vs. Low MAP during cardiopulmonary bypass in order to decrease morbidity and mortality associated with CABG surgery [13]. At the time this study was conducted (1991–1994), MAP during cardiopulmonary bypass was routinely maintained at 50–60 mm Hg and believed to be safe. The results of this study provided the first evidence that lower blood pressure (Low MAP) was associated with higher rates of cardiac and neurologic complications in the setting of CABG surgery, and moreover, that High MAP during CABG could both protect against cardiac and neurologic complications [13] and be done safely [16]. Optimal blood pressure management during CABG surgery is still debated 20 years later [23,24]. However, it is now clear that high risk CABG surgery patients require increased perfusion pressures (High MAP) while on cardiopulmonary bypass, and MAPs during CABG surgery are maintained higher today than they were 20 years ago [23,25].

### Advantages and disadvantages to Home F/U vs. Routine F/U

The most obvious advantage of the Home F/U approach is enhanced follow-up of participants, resulting in decreased selection bias (Tables 3 and 4). An alternative to Home F/U to reach sample size is to continue enrolling new participants to replace participants who have not returned for follow-up or who have dropped out. However, there is extensive evidence that lost participants are qualitatively different than participants who complete study follow-up. For example, people who do not complete follow-up are more likely to have sustained adverse outcomes, died or have worse health and function [1-3]. We found that people with 2 or more complications were more likely to require Home F/U. Without Home F/U, the complications sustained by these participants (8.1%, see Table 6) would not have been counted in our study results. Home F/U does have disadvantages, including time intensity of the approach and cost. Another potential disadvantage may be that the way participants answer study questions might be different at home compared to in the hospital setting, which is germane to the neurocognitive tests and quality of life assessment (e.g. SF-36), but not assessment of clinical complications.

In multivariate analysis, we found that Home F/U participants had different demographic and clinical characteristics when compared to Routine F/U participants – at baseline they were more likely to be older and receiving diuretic therapy. Of the participants on diuretics, 70% did not have a history of CHF. We hypothesized that because of their older age and medical illnesses requiring diuretic therapy (e.g., refractory hypertension), Home F/U participants were more likely sedentary with other non-life-threatening chronic illnesses (conditions not assessed by the Charlson Index, which only assesses conditions that increase mortality risk) that made returning to the hospital for 6-month follow-up difficult (e.g., osteoarthritis).

Taken collectively, the most common predictors of drop out are older age, cognitive dysfunction and functional impairment. Older age is the most common factor associated with loss to follow-up [26-30]. We found that participants who were older were more likely to require Home F/U, particularly participants who were 75 years of age or older (OR=3.23, 95% CI 1.52-6.88). Cognitive impairment is also a well-recognized predictor of loss to follow-up [26]. Blumenthal found that cardiac surgery patients who dropped out of a study of neurocognitive functioning over time had lower baseline neurocognitive scores when compared to those who completed 6-week and 6-month evaluations [31]. We similarly found that Home F/U participants scored lower at baseline on the Ammons IQ test, the Benton recognition test, Trails B and Digit Symbol (Table 1). However, neurocognitive function was not a significant predictor of the need for Home F/U in our multivariate model (Table 7).

Participants who drop out of epidemiologic studies have worse health and function. Mihelic and Crimmins reported that having difficulty with activities of daily living was predictive of drop out in an epidemiologic study of older adults [28]. Markides [27] reported that drop outs had lower self-rated health. Our study supports this, as evidenced by the finding that Home F/U added 4 additional functional complications. In addition, we found that participants requiring Home F/U were more likely to have sustained 2 or more major complications when compared to Routine F/U participants (p=0.05) (Table 6). Finally, participants on diuretics prior to surgery were 2.4 times more likely to require Home F/U at 6 months (OR=2.44, 95% CI, 1.14-5.21).

### Limitations

Our study had several limitations. First, this RCT was conducted 20 years ago and the cardiac surgery population may be different now. Nonetheless, we believe that the methodologic bias introduced as a result of drop outs is a timeless issue and that Home F/U remains a viable strategy to achieve follow-up for missing participants in contemporary clinical research, particularly because cardiac surgery patients are now older, with greater comorbidity [9], which our findings suggest increase the risk for attrition. Second, this was not an RCT of Home F/U vs. Routine F/U. Therefore, the study was not powered to examine the timing of complications relative to potential loss to follow-up or formally establish clinical predictors of participants who required Home F/U. Nonetheless, the study demonstrates the methodologic importance of follow-up on RCT results and the feasibility of Home F/U as a strategy to reduce attrition. Third, responses to assessments may be different in the patient’s home compared to what they might have been in the hospital setting, but, as mentioned earlier, this would only affect subjective measures.

## Recommendations

We offer several recommendations to help prevent drop out. Given the time and resources required to conduct Home F/U, we have developed a number of strategies to retain participants. We believe that by employing these approaches, we decrease the likelihood that we will lose participants over the course of a longitudinal study.

1. Inform participants of the study requirements and ensure they are aware of the follow-ups that are required [32].

2. Obtain alternate contacts; we recommend at least 2, preferably 3, alternates who do not reside with the participant.

3. Study personnel should strive to develop positive, friendly relationships with participants, be helpful and accommodating, allocate adequate time for follow-ups, and schedule follow-ups on days and at times that are convenient for participants, including evenings and weekends.

4. Limit follow-up evaluations in length and frequency. Schedule study follow-ups to coincide with other routinely scheduled clinical appointments whenever possible.

5. Refusal of 1 follow-up is not necessarily a dropout. Moreover, when participants are at risk for withdrawal, it is important to ascertain the reason. Studies have found that life events, such as divorce or retirement, may lead to study drop out [33]. It is possible that participants will consent to being contacted in the future, once the personal situation has resolved [34].

6. Stay alert for participants who are “at risk” for dropping out (e.g., failure to return calls, missing appointments) [34]. If a participant refuses to return, consider employing alternate sources of information to ascertain clinical status (e.g., clinical notes). If a participant does drop out, record detailed reasons. Another alternative that has been suggested is triggered sampling, where additional information is collected when a designated health marker declines, but prior to drop out [35].

7. Create an “essential evaluation” that has only questions that are most critical to your study, which can be employed with participants who are at high risk of dropping out.

8. Finally, offer Home F/U to those participants who are at risk of dropping out, particularly when assessing final study complications.

Our study differs from other studies that have examined attrition. Because the participants have dropped out in other studies, the participant’s health status with respect to the study complications is unknown when they have dropped out. In contrast, the current study presents a mechanism to decrease attrition and obtain follow-up on participants who would likely have dropped out of the study. In this manner, we are able to add to the literature and report participant health status at the point in time when they were at high risk for dropping out of the study. These results were obtained in the context of a study of CABG surgery patients. However, we believe these results are generalizable to other patient groups, and Home F/U should be considered a feasible approach to decrease study attrition in longitudinal clinical research studies. We recommend that intervention studies of clinical populations consider home visits as a viable mechanism to decrease attrition and improve longitudinal follow-up.

## Conclusions

We have demonstrated that Home F/U is a feasible approach to achieve 4% loss to follow-up in a longitudinal clinical trial. We found that the timing of complications (early vs. late) was not related to the need for Home F/U, but participants who sustained 2 or more complications were significantly more likely to require Home F/U. In addition, we found that participants in the Home F/U group were more likely to be 75 years or older and on diuretic therapy at baseline, indicating that these participants were both older and more medically ill at baseline. The data that we examined for this paper was from a study of CABG surgery patients conducted 20 years ago and this was not a randomized comparison of Routine F/U vs. Home F/U. Nonetheless, over the intervening years, the issue of loss to follow-up remains an ongoing and critically important methodological challenge in clinical research. While new statistical procedures have been developed to impute missing data over the intervening years, there is no replacement for actual clinical data obtained from participants. Finally, we have offered recommendations to decrease the likelihood of loss to follow-up over the course of a longitudinal study.

## Abbreviations

Home F/U: Home visit to obtain final study evaluation at 6 months; MAP: Mean arterial pressure; RCT: Randomized controlled trial; CABG: Coronary artery bypass graft; Low MAP: Usual care, mean arterial pressure during cardiopulmonary bypass maintained at 50–60 mm Hg; High MAP: Experimental group, mean arterial pressure during cardiopulmonary bypass maintained at 80–100 mm Hg; CHF: Congestive heart failure.

## 

Supported by NHLBI R01 HL44719.

## Competing interests

All authors declare that they have no competing interests.

## Authors’ contributions

JP conceived of the study, participated in the acquisition and interpretation of data, performed the statistical analysis, drafted the manuscript and approved the final version to be published. PP participated in the acquisition and interpretation of data, critically revised the manuscript for important intellectual content and has given final approval of the version to be published. MC participated in the acquisition and interpretation of data, critically revised the manuscript for important intellectual content and has given final approval of the version to be published. MW participated in the analysis and interpretation of data, critically revised the manuscript for important intellectual content and has given final approval of the version to be published.

## Authors’ information

**Janey C. Peterson EdD, MS, RN** is Assistant Professor of Clinical Epidemiology in Medicine, Integrative Medicine and Cardiothoracic Surgery at Weill Cornell Medical College. Dr. Peterson is a clinical epidemiologist, behavioral scientist and health educator who has worked in critical care and clinical trials for over 20 years, evaluating the efficacy of clinical and behavioral interventions in older adults with a wide variety of chronic medical conditions, including patients with cardiovascular disease, breast cancer, homebound elderly, general medicine outpatients, neurosurgical patients and cardiac surgery patients. Over the past several years, her work has focused on promoting behavior change in older adults with chronic illness. Dr. Peterson's methodological expertise is in trial design and measurement. She currently serves as Chair of two NIH Committees that focus on Safety and Data Monitoring and Recruitment and Retention.

**Paul A. Pirraglia, MD, MPH** is Acting Chief of Primary Care at the Providence VA Medical Center and an Assistant Professor of Medicine at the Warren Alpert Medical School of Brown University. Dr. Pirraglia has a distinguished research and teaching background. He was the recipient of a VA Health Services Research & Development Career Development Award from 2005–2008 for research on the overlap of chronic obstructive pulmonary disease with depression and anxiety, and he continues to do research on providing care at the intersection of physical and mental health. He is a strong proponent of primary care, serving as the New England Regional President of the Society of General Internal Medicine from 2009 to 2010. He teaches medical students and residents in the clinical setting, and precepts the VA Journal Club.

**Martin T. Wells, PhD** is Chair of the Department of Statistical Science at Cornell University, with joint appointments in the Department of Biological Statistics and Computational Biology, the Department of Social Statistics and the Division of Clinical Epidemiology and Health Services Research at the Weill Cornell Graduate School of Medical Sciences. He is interested in methods development for analyzing clinical and biological data. He is currently an Editor of the *Journal of Multivariate Analysis* and *Journal of Empirical Legal Studies.* He was previously an Editor of the *Journal of American Statistical Association, Statistical Science* and served as the Editor-in-Chief of the *ASA-SIAM Book Series.* Additionally, he was a member of the National Academy of Science Advisory Panels to Evaluate Census 2000, the American Statistical Association’s Advisory Committee to the Bureau of Justice Statistics. He is also a Fellow of the American Statistical Association, Institute of Mathematical Statistics and the Royal Statistical Society and is an Elected Member of the International Statistical Institute.

**Mary E. Charlson, MD** is Professor of Medicine and Chief of the Division of Clinical Epidemiology and Evaluative Sciences Research at Weill Cornell Medical College. Dr. Charlson is a methodologist and nationally recognized expert in measurement of prognostic risk. She developed the Charlson Comorbidity Index, the most commonly used prognostic measure of illness burden in contemporary clinical research [14]. Dr. Charlson is the director of the Agency for Health Care Research and Quality (AHRQ) T32 training program in Clinical Epidemiology and Health Services Research at Weill Cornell Medical College and she is the director of the Master’s program in Clinical Epidemiology and Health Services Research. She has trained over 40 investigators who have gone on to successfully compete for independent funding. As a clinical epidemiologist, her work has focused on developing prognostic models for use in patients with acute and chronic illnesses and developing and testing interventions targeted at secondary prevention of disease. She has been funded by multiple NIH RO1’s, an NHLBI Consortium Contract Award and is currently conducting a multi-phase U01 award, also funded by NHLBI.

## Pre-publication history

The pre-publication history for this paper can be accessed here:

http://www.biomedcentral.com/1471-2288/12/178/prepub
